# Intravenous Transplantation of Mesenchymal Stromal Cells to Enhance Peripheral Nerve Regeneration

**DOI:** 10.1155/2013/573169

**Published:** 2013-12-26

**Authors:** Stella M. Matthes, Kerstin Reimers, Insa Janssen, Christina Liebsch, Jeffery D. Kocsis, Peter M. Vogt, Christine Radtke

**Affiliations:** ^1^Department of Plastic, Hand and Reconstructive Surgery, Hannover Medical School, 30659 Hannover, Germany; ^2^Department of Neurology and Center for Neuroscience and Regeneration Research, School of Medicine, Yale University, New Haven, CT 06510, USA; ^3^Rehabilitation Research Center, Veterans Affairs Connecticut Healthcare System, West Haven, CT 06516, USA

## Abstract

Peripheral nerve injury is a common and devastating complication after trauma and can cause irreversible impairment or even complete functional loss of the affected limb. While peripheral nerve repair results in some axonal regeneration and functional recovery, the clinical outcome is not optimal and research continues to optimize functional recovery after nerve repair. Cell transplantation approaches are being used experimentally to enhance regeneration. Intravenous infusion of mesenchymal stromal cells (MSCs) into spinal cord injury and stroke was shown to improve functional outcome. However, the repair potential of intravenously transplanted MSCs in peripheral nerve injury has not been addressed yet. Here we describe the impact of intravenously infused MSCs on functional outcome in a peripheral nerve injury model. Rat sciatic nerves were transected followed, by intravenous MSCs transplantation. Footprint analysis was carried out and 21 days after transplantation, the nerves were removed for histology. Labelled MSCs were found in the sciatic nerve lesion site after intravenous injection and regeneration was improved. Intravenously infused MSCs after acute peripheral nerve target the lesion site and survive within the nerve and the MSC treated group showed greater functional improvement. The results of study suggest that nerve repair with cell transplantation could lead to greater functional outcome.

## 1. Introduction

Common causes of disastrous nerve injuries include motor vehicle accidents, violence, sports-related injuries, and falls [1]. Traumatic nerve damages can lead to complete functional loss of the affected limb and are often combined with life threatening injuries which have to be treated first. During this time, the transected nerves undergo Wallerian degeneration [2] in parallel to irreversible muscle degeneration. After peripheral nerve injury, the duration of nerve transection before reinnervation of effected organ is critical; even after immediate nerve repair, clinical results are often disappointing. Therapeutic strategies to improve and especially accelerate axonal regeneration and remyelination are of great importance.

Cell-based therapies using mesenchymal stromal cells (MSCs) are being investigated in clinical trials for a number of neurological diseases including stroke [3] and peripheral nerve [4] and spinal cord [5] injuries. The rationale is that the transplanted MSCs provide neuroprotection, neovascularisation, and induction of axonal sprouting by their production of cytokines and neurotrophic factors [6]. Peripheral myelin-forming cells (Schwann cells and olfactory ensheathing cells) have been shown to improve survival when directly transplanted into peripheral nerve and lead to improvement in functional outcome [7–9]. However, harvesting of these cells requires nerve biopsy in the case of Schwann cells and biopsy from nasal mucosa both of which have some potential morbidity associated with them. A major issue preventing clinical use of OECs for intralesional cell transplantation after nerve injury is the difficulty to harvest a sufficient amount of viable autologous cells in the injured individual. Resulting donor site morbidity such as impairment of smell or anosmia may limit clinical use. The harvesting of bone marrow derived MSCs in patients is a common procedure and has low morbidity, thus making these cells attractive as potential cell transplantation source.

While a relatively large number of experimental and clinical studies have been carried out with direct or intravenous infusion of MSCs (see [3, 6] for review) the repair potential of intravenously transplanted MSCs in peripheral nerve injury has not been addressed. The primary objective of this study was to determine if intravenously transplanted MSCs following peripheral nerve transection reach the lesion site and what impact the MSCs have on functional recovery.

## 2. Methods

### 2.1. Cell Preparation

MSCs were prepared as previously described with modifications [10, 11]. Cells were prepared from bone marrow aspirates (10 *μ*L), which were isolated from femur and tibia of adult rats using a heparinized 24G needle. Cell material was diluted 1 : 1 with *α*-MEM (Invitrogen, Karlsruhe, Germany) and filtered through a 70 *μ*m nylon mesh (Cell Strainer, BD Falcon; Becton Dickinson, Franklin Lakes, NJ, USA). The resulting cell suspension was layered on top of 15 mL Ficoll-Paque Plus (Amersham Pharmacia Biotech, Uppsala, Sweden) and centrifuged for 30 min at 800 ×g at room temperature. The supernatant and interface were combined, diluted to about 50 mL with PBS (0.1 M) and centrifuged for 10 minutes at 800 ×g. After discarding of the supernatant the pellet was resuspended in 1 mL medium. The nucleated cells were counted and suspended at a concentration of 1 × 10^7^/mL in the growth medium (*α*-MEM) supplemented with 2 mg/mL L-glutamine, 50 *μ*g/mL streptomycin, and 20% (v/v) of not heat-inactivated fetal calf serum) and plated at 3 × 10^6^/cm^2^ in 100 mm culture dishes (Falcon, Becton Dickinson). The cells were incubated for 3 days, and the nonadherent cells were removed by replacing the medium in three washing steps. After the cultures reached confluency, the cells were dislodged by incubation with Accutase (PAA, Cölbe, Germany) at 37°C for 3-4 min. They were diluted and replated at a density of 2000 cells/cm^2^ in 100 mm culture dishes. Cells were used for transplantation after 7 days of cultivation. Immunostaining of the cells with an anti-CD-90 antibody (monoclonal mouse antibody, Abcam, Cambridge, UK, 1 : 800), Stro-1, and CD 44 demonstrated that the purity of the BM-MSC preparations was >90% (data not shown). The cells did not stain for CD34 and CD45.

### 2.2. Nerve Lesion and Cell Transplantation Procedure

All animal experiments were approved by the Lower Saxony district government and the Medical School of Hannover and conducted according to the German Law of Animal Protection and were performed in accordance with National Institutes of Health guidelines for the care and use of laboratory animals, and the Veterans Affairs Connecticut Healthcare System Institutional Animal Care and Use Committee approved all animal protocols respectively.

Adult wild Sprague Dawley rats (200–225 g) were used for this experiment (*n* = 18). The rats were anesthetized with ketamine (75 mg/kg i.p.) and xylazine (10 mg/kg i.p.). Preoperatively the rats underwent a splenectomy. The sciatic nerve was surgically exposed in anesthetized rats and completely sectioned by standardized nerve crushing the level of the piriformis tendon in the thigh. Cultured MSCs were detached from the culture flasks and resuspended in culture medium and prelabelled with PKH26. Using a syringe, 2 mL of the cell suspension, vehicle alone or negative control cells (fibroblasts), were injected via femoral vein directly after lesion induction. The animals survived for 21 days followed by scarification with removal of nerves for histological analysis.

### 2.3. Footprint Analysis

Determination of the walking track with analysis of the sciatic functional index (SFI) was performed according to the method described by De Medinaceli et al. [12]. To obtain footprints, hind paws were placed on an ink blotter and the animals were placed on a white piece of paper. Both feet produced five to six prints. Rats were tested weekly over the course of the experiment. Surgery was done on the left sciatic nerve of each animal and the right hind limb was used as internal control. Footprints were collected from the experimental (E, left) and normal (N, right) sides. Prints were measured for the following parameters: distance between foot prints (TOF), the entire plantar length (PL), the distance from the first to fifth toes, the toe spread (TS), the distance between the second and fourth toes, and the intermediary toe spread (IT). The SFIwas calculated according to the following formula:

(1) (ETOF−NTOF)NTOF+(NPL−EPL)EPL  +(ETS−NTS)NTS+(EIT−NIT)NIT×2204.

Calculated indices from this formula ranged between a score from zero to minus 100. Zero describes normal function and −100 complete transection of the sciatic nerve. Data are presented as means±SE. Statistical evaluations were based on two-tailed *t*-test and *χ* ^2^ test (origin; criterion, *P* < 0.05).

### 2.4. Immunohistochemistry

Sciatic nerves from transplanted and control rats were processed for immunocytochemistry as described previously. Briefly, rats were deeply anesthetized with ketamine/xylazine and perfused transcardially, firstly with 0.9% saline and then with ice-cold 4% paraformaldehyde in 0.14 M Sorensen's phosphate buffer, pH 7.4. Sciatic nerves were removed and postfixed for 20 min in 4% paraformaldehyde. Tissue was then cryoprotected in 30% sucrose in 0.14 M Sorensen's phosphate buffer overnight at 4°C. Ten micrometer longitudinal cryosections of the sciatic nerves were cut and mounted on Silane Prep glass slides (Sigma, St. Louis, MO, USA). Sections were processed for immunostaining for monoclonal antibody neurofilament (NF, Sigma, St Louis, MO, USA; dilution 1 : 1000) followed by incubation with secondary antibody goat anti-mouse IgG-Alexa Fluor 594 (Invitrogen, Eugene, OR, USA; 1 : 1000) and coverslipped with DAPI-containing mounting media (VectaShield, Vector Laboratories, Burlingame, CA, USA). The sections were examined with a fluorescence microscope (Nikon Eclipse 800; Spot RT Color CCD camera; Diagnostic Instruments).

## 3. Results

### 3.1. PKH 26 Labelling and Crush Lesion

**Figure 1:**
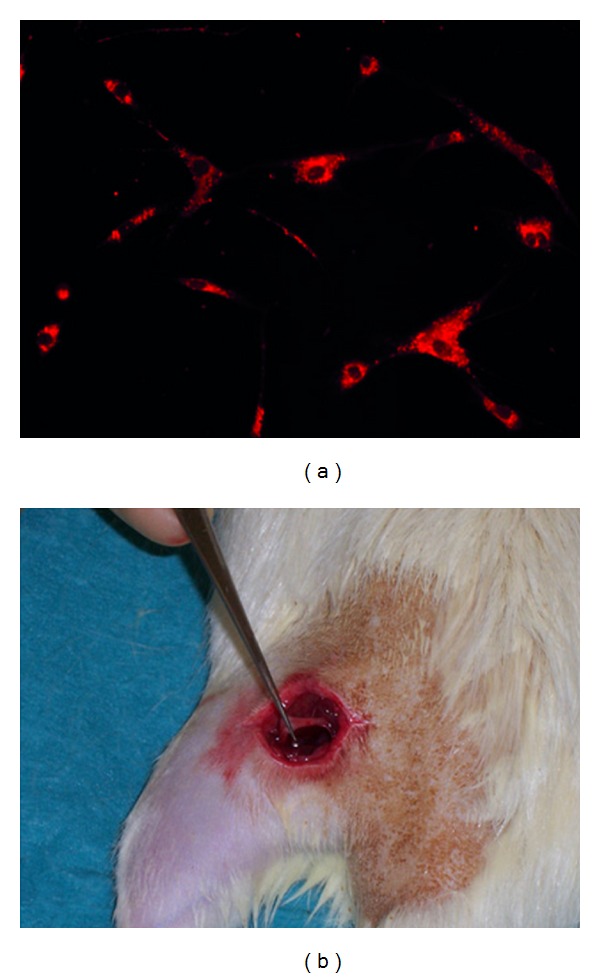
Prelabelling of cells and of intravenous injection via femoral vein. Cultured MSCs were stained with the membrane dye PKH26 before cell transplantation (a). Sciatic nerve lesion was induced by nerve crush (b), followed by intravenous injection of MSCs via femoral vein.

The sciatic nerve was surgically exposed in anesthetized rats and its axons were transected by nerve crush (Figure 1(b)). The transection site was standardized at the level of the piriformis tendon in the thigh. Isolated MSCs in culture showed characteristic flattened fibroblast-like morphology after cell attachment and removal of nonadherent cells. MSCs were immunopositive for the stem cell markers CD 90, CD 44, and Stro-1 and were immunonegative for the hematopoietic stem cell markers CD 34 and CD 45 (data not shown). Immediately before cell transplantation, cultured MSCs were detached from the culture flasks and resuspended in serum free culture medium and prelabelled with PKH26 (Figure 1(a)) for in vivo cell tracing after injection.

For intravenous systemic injection (*n* = 18), animals received MSCs (1.0 × 10^6^) in 1 mL total fluid volume (DMEM) of the cell suspension or vehicle alone (sham control; *n* = 6) by using a syringe. The intravenous cell injection was performed via the femoral vein.

**Figure 2:**
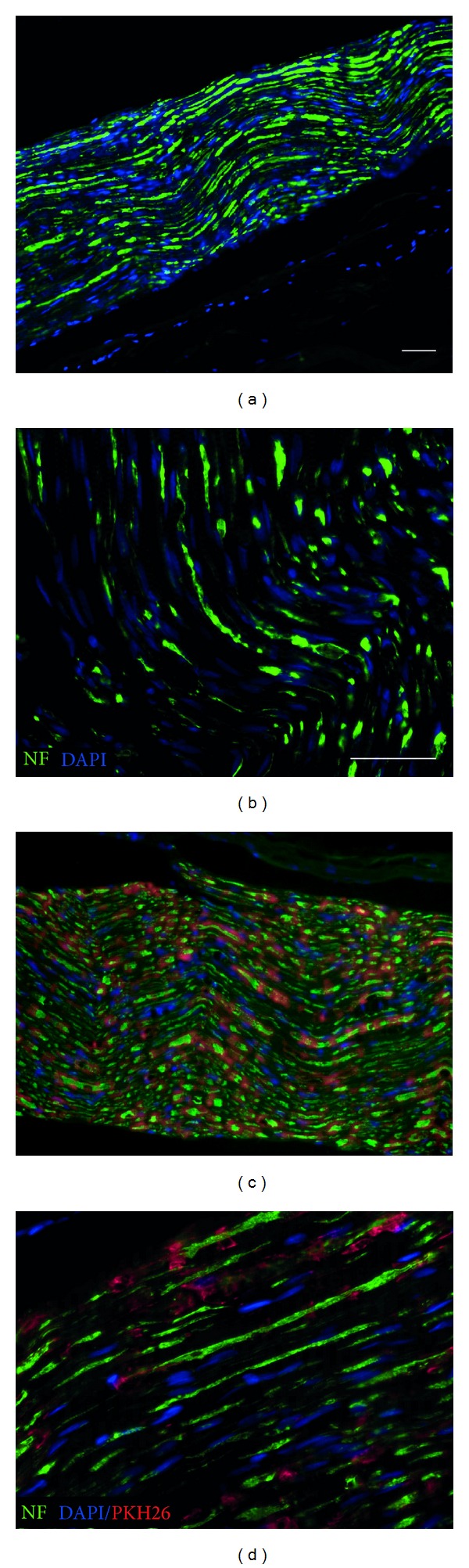
Longitudinal sections and immunohistology of sciatic nerve after peripheral nerve crush injury and intravenous transplantation of PKH-labelled MSCs. (a) and (b) Demonstration of axonal regeneration after peripheral nerve injury and cell transplantation: regenerated axons are stained with neurofilament (green) in low and higher magnifications. (c) and (d) Distribution of i.v. transplanted PKH-labelled MSCs (red) and homing effect into the lesioned and subconsequent regenerated nerve fibers in relation to the neurofilament stained axons and endogenous cells stained with DAPI present within the nerve (blue).

Three weeks after lesion induction and systemic cell delivery, the nerves were removed and prepared for histology. In both experimental groups there was evidence of increased axonal regeneration and improved functional outcome. However, the MSC transplantation group (Figures 2(c) and 2(d)) in comparison to the control group in low and high power images (Figures 2(a) and 2(b), resp.) had greater numbers and more axons proximal, within and distal to the repair site. In frozen sections of the nerve, PKH26-labelled MSCs could be found within the regenerated peripheral nerve after systemic delivery shortly after lesion induction indicating a homing effect of the MSCs to the peripheral nerve lesion site (Figures 2(c) and 2(d)). The MSCs (red) survived in the lesion site and distributed longitudinally across the lesion site in both proximal and distal directions with the regenerated axons. The regenerated axons are stained with neurofilament (green) in low and higher magnifications (Figures 2(a)–2(d)). The extended distribution of the transplanted PKH-labelled MSCs (red) demonstrates the homing effect into the lesioned peripheral nerve and subsequent regenerated nerve fibers in relation to the neurofilament stained axons (Figures 2(c) and 2(d)).

### 3.2. Functional Analysis of Stepping Behaviour

**Figure 3:**
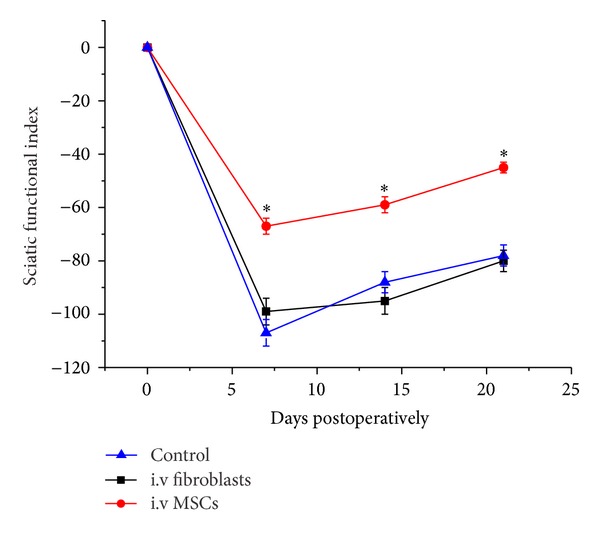
Foot print analysis after intravenous cell transplantation after sciatic crush lesion. The animals with MSC transplanted nerve showed greater functional recovery as scored using the Sciatic Functional Index (SFI) than in sham control or control cell transplantation indicating that the cells have an enhancing effect for axonal regeneration and remyelination which results in improved functional outcome. Data are presented as means ± SE. Statistical evaluations were based on two-tailed *t*-test, *χ* ^2^ test (Origin; criterion, *significantly different, *P* < 0.05).

Footprint analysis using the sciatic nerve functional index (SFI) was carried out before lesion (day 0), beginning seven days after nerve crush and i.v. cell injection (MSCs or fibroblasts) and sham control (media infusion). The SFI score is zero for normal animals and a negative value for nerve impairment. At day seven the MSC group showed greater functional improvement (66.25 ± 3.75) than either the control cell injection (fibroblast) or the sham (vehicle alone) groups (98.8 ± 4.4 versus 106.0 ± 4.5). The locomotor improvement was observed in 14 days (MSCs 58.0 ± 3.0 versus fibroblast cell injection 86.2 ± 3.95 and vehicle alone 94.9 ± 3.85) and maintained in 21 days (MSCs 44.7 ± 2.5 versus fibroblast cell injection 77.5 ± 4.5 and vehicle alone 79.9 ± 4.0; see Figure 3). The functional improvement in the MSC group was significant in 7 days, but the rate of change in improvement from 14 to 21 days was comparable between the three experimental groups, suggesting that the effect of the MSCs was an early interventional event.

## 4. Discussion

The primary objective of this study was to investigate whether systematically administered MSCs target a site of a nerve crush lesion and lead to functional improvement. To this end MSCs were isolated from isogenetic rats and labelled with PKH26 which is known to have a long half-life in vivo and has been successfully used in experimental neurological settings [13]. PKH26-labelled cells were found in the regenerated nerves three weeks after the transplantation demonstrating that at least part of the transplanted cells integrated into the lesion site where they associated with regenerating nerve fibers. Interestingly, labelled negative control cells were not found in the lesion site. Functional recovery in the MSCs transplanted animals as assessed using footprint analysis was significantly increased compared to untreated base levels and fibroblast transplanted negative cellular controls.

Using MSCs expressing firefly luciferase it has been demonstrated that MSCs selectively are incorporated into sites of inflammation such as cutaneous wounds and tumors while there was a bioluminescence clearance over 14 days in uninjured, nontumor-bearing animals [14]. It has been proposed that this tropism is due to inflammatory chemokines and hypoxic conditions in the respective microenvironment (reviewed in [15]). Intravenously injected MSCs have also been demonstrated to be incorporated into demyelinated and traumatic spinal cord injuries in rats where they induced a significant clinical improvement [11, 13]. Functional improvement after delivery of MSC has also been reported in several preclinical and clinical studies dealing with neurodegenerative diseases (e.g., reviewed in [16]). A recent study found structural changes and improved functional outcome following MSC infusions in a spinal cord injury model in the rat but did not detect MSCs in the lesion site [17]. Significant functional improvement was also observed following intravenous infusion of MSCs in a myocardial infarction model [18]. Interestingly, only a limited number of MSCs reached the lesion site and the vast majority of cells lodged in the lungs with a half-life of 24–48 hours. The MSCs were shown to produce the powerful multipotent anti-inflammatory compound and tumor necrosis factor-inducible gene 6 protein (TSG-6). Infusion of TSG-6 knock-down MSCs was much less efficacious. Thus, a potential mode of action of intravenous infusion of MSCs could be the “remote” production of anti-inflammatory or trophic factors in peripheral organs such as lung which then exert a systemic effect. These critical issues on the mechanism of action of MSCs require additional research.

Several biological characteristics of MSCs as applied to regenerative medicine [18] may contribute to enhanced neuronal regeneration. First, MSCs secrete a number of immunomodulatory factors [19, 20]. An appropriate inflammatory response is necessary to prevent infection and to start the repair process by removing myelin and cell debris, but extensive inflammation can also have negative influence [21]. As a second measure MSCs also provide angiogeneic factors stimulating an appropriate vascularization of the regenerating tissue [22]. Vascular damage and resulting undersupply of nutrients and oxygen often accompany peripheral nerve injuries. Administration of vascular endothelial growth factor in a nerve conduit used to bridge a 1 cm sciatic nerve defect led to enhanced axonal regeneration with 78% more myelinated axons after 180 days [23].

## 5. Conclusion

In summary our results indicate that intravenous MSCs can improve functional outcome in a peripheral nerve injury model and that some of the transplanted reach and survive in the lesion area for at least three weeks. The precise mechanism for this beneficial effect is not yet clear, but a number of mechanisms including immunomodulation, stimulating neovascularization, and neurotrophic influences may contribute. Given that intravenous infusion of MSCs have been used in a number of clinical studies with demonstrated safety (see [6]) the prospect of using MSCs as an adjunct therapy in nerve defect repairs should be considered.
